# A Digital Health Program Targeting Physical Activity Among Adolescents With Overweight or Obesity: Open Trial

**DOI:** 10.2196/32420

**Published:** 2022-03-28

**Authors:** Caroline Cummings, Rebecca Crochiere, Amy Hughes Lansing, Riya Patel, Catherine Stanger

**Affiliations:** 1 Department of Psychological Sciences Texas Tech University Lubbock, TX United States; 2 Department of Psychological and Brain Sciences Drexel University Philadelphia, PA United States; 3 Department of Psychological Science University of Vermont Burlington, VT United States; 4 Geisel School of Medicine Dartmouth College Hanover, NH United States

**Keywords:** mHealth program, physical activity, adolescent overweight, adolescent obesity, incentives, mobile phone

## Abstract

**Background:**

Prior studies suggest that mobile health physical activity programs that provide only weekly or daily text-based health coaching evidence limit the efficacy in improving physical activity in adolescents with overweight or obesity. It is possible that incentives, combined with health coaching and daily feedback on goal success, may increase program efficacy; however, such programs have not yet been tested with adolescents with overweight and obesity.

**Objective:**

This study aims to examine the feasibility and acceptability of a 12-week, incentive-based, mobile health physical activity program with text-based health coaching, goal setting, and self-monitoring for adolescents with overweight or obesity. Program adherence and changes in tracked physical activity (ie, steps and active minutes while wearing a Fitbit [Google LLC]), body mass, and body fat are assessed.

**Methods:**

A total of 28 adolescents aged 13 to 18 years with a BMI ≥90th percentile participated in the program. Of the 28 participants, 2 (7%) were lost to follow-up; thus, data from 26 (93%) participants were used in analyses.

**Results:**

Participant-reported acceptability was high, with all mean ratings of text-based coaching, Fitbit use, and the overall program being >5 on a 7-point scale. In addition, 85% (23/26) of participants reported that they would like to continue to wear the Fitbit. Program adherence was also high, as participants wore the Fitbit on 91.1% (SD 12.6%) of days on average and met their weekly goals for an average of 7 (SD 3.5) of 11 possible weeks. There were no demographic (ie, sex, age, and baseline body mass) differences in the percentage of days participants wore their Fitbit. Across the 12-week study, there were significant improvements in tracked daily active minutes (*P*=.006) and steps (*P*<.001) and significant pre- to posttest improvements in body fat percentage (*P*=.04).

**Conclusions:**

The pilot program improved adolescent physical activity and physical health. A larger factorial design trial with adaptive daily goals may clarify the role of each program component in driving physical activity.

## Introduction

Adolescence is a high-risk period to have overweight and obesity [1], which may be in part attributable to a decline in physical activity found in this age group. Addressing having overweight and obesity during adolescence is imperative to reduce the risk of continued overweight and obesity—and their common comorbid health conditions—into adulthood [2]. Fewer than 40% of adolescent girls and 30% of adolescent boys meet physical activity recommendations, yielding <10,000 steps per day or 60 minutes of moderate to vigorous physical activity [3]. Traditionally, physical activity intervention programs targeting overweight or obesity among adolescents include in-person, scheduled, activity-based intervention programs to support physical activity habit formation and demonstrate notable downstream improvements in body fat [4] and equivocal efficacy in improving body mass [5-7]. However, despite the general successes of physical activity intervention programs, adolescents with overweight or obesity and their families struggle with accessing and completing such intervention programs, limiting treatment benefits [8,9]. The two major reasons for attrition are a lack of motivation or interest in increasing physical activity and a lack of time to dedicate to participating in a physical activity intervention [10]. Thus, there is a need to test highly scalable intervention programs designed to promote physical activity in adolescents with overweight and obesity, which may also produce secondary effects on body mass and body fat. Importantly, considerations of how to motivate physical activity change must be made during the program development process to reduce the risk of attrition. This study included the pilot-testing of a mobile health (mHealth) program that incentivizes an increase in tracked physical activity in adolescents with overweight or obesity. Program acceptability, program adherence, and changes in physical activity (ie, increase in tracked active minutes and steps) were assessed. A series of exploratory analyses assessed possible changes in body mass and body fat from baseline to after the intervention.

mHealth physical activity intervention programs can reduce the cost and time burden for participants [11], and the inclusion of electronic feedback with goal setting may further improve intervention effects. mHealth programs are uniquely suited to improve adolescent health behavior, as a high percentage of adolescents have access to mobile technologies and report a preference for mHealth programs [12]. Within mHealth physical activity programs, specifically, there is a reliance on an array of remote components, including passive sensing to track behavior (eg, momentary feedback via a pedometer) [13-15] and text-based coaching with goal setting [16,17]. Unfortunately, mHealth physical activity programs that provide only weekly or daily text-delivered messages to adolescents evidence limited efficacy in improving physical activity [16,17]. In contrast, in adult samples, physical activity–promoting mHealth programs that use adaptive goal setting and incentives are efficacious in increasing physical activity [13]. In addition to electronic tracking and feedback, the application of incentives within an mHealth program may further enhance physical activity goal achievement in adolescents with overweight and obesity.

Incentives may be an effective method of increasing engagement in health behaviors in adolescents [18], and incentives for meeting adaptive goals may support improved physical activity in adolescents with overweight and obesity. Incentives that are tied to an individual’s success in meeting a specific behavioral goal are hypothesized to be a more effective method than incentives delivered for intervention participation alone [18]. Incentives have been effective in facilitating health behavior change in adolescents with chronic health conditions, such as type 1 diabetes [19,20]. A recent meta-analysis found that incentives also promote improved physical activity goal achievement for adults participating in a physical activity intervention program [21]. On the basis of behavioral principles (ie, operant conditioning), researchers theorize that incentives support immediate improvements in physical activity through the use of positive reinforcement (ie, provision of monetary rewards) that is provided with high frequency (ie, daily or weekly) and predictability (ie, always after a set number of times the individual meets the physical activity goal) [22]. Moreover, the provision of incentives for meeting adaptive rather than static goals has been shown to yield the greatest improvements in physical activity [13], although a decline in activity may be found in the later phases of an intervention [23]. Therefore, to generate robust physical activity change that persists across a program, there may need to be two key features—(1) a shaping phase to incrementally establish a physical activity habit by providing immediate rewards in response to meeting increasingly challenging behavioral goals and (2) continued support for short-term and long-term preservation of habitual physical activity engagement through providing immediate rewards for continued goal success (ie, maintenance phase) and then delayed rewards for continued goal success (ie, fading phase) [24]. However, the provision of incentives in response to physical activity goal success within a scalable, mHealth physical activity program has not yet been tested with a sample of adolescents with overweight and obesity.

This manuscript describes the pilot-testing of a 12-week mHealth incentive program, which targets increased physical activity in adolescents with overweight or obesity. This study is a single group, pre–post pilot of a physical activity program with fixed daily and adaptive weekly physical activity goals, incentives for meeting goals, daily text-based feedback about goal success, and weekly text-based supportive coaching. The primary aim is to examine program acceptability (ie, perceived helpfulness of the text-based coaching, enjoyment of Fitbit (Google LLC) use, and overall program impressions). Secondary aims are to examine (1) program adherence (ie, goal attainment and incentive earnings), including demographic factors related to Fitbit use, and (2) changes in physical activity (ie, increase in tracked active minutes and steps). Finally, an exploratory aim is to identify the possible benefits of this program on body mass and body fat.

## Methods

### Participants

Participants included (N=28) adolescents with overweight or obesity (15/27, 54% male; mean age 14.81, SD 1.59; mean BMI percentile 97.07, SD 1.85; 26/27, 93% White). Most participants (21/27, 78%) reported having private health insurance. Inclusion criteria included the following: age 13 to 18 years; BMI percentile >90; having broadband wireless internet at home; living at home; and permission from the pediatrician to engage in a physical activity program (eg, physical education or sports), which was noted in electronic health records. Although the 85th and 95th percentile are the standard cutoffs for having overweight and obesity [25], respectively, criteria were modified to include those participants who had obesity or who were at least at moderate or high risk of obesity (ie, at or above the top half of the BMI percentile range for having overweight). Participation in this study also included neuroimaging; thus, exclusion criteria included the following: contraindicated metallic objects in their bodies, psychiatric medication, pregnancy, neurological or health problems (other than obesity), morbid obesity that prevented entering scanner, and visual acuity that could not be corrected to normal.

### Ethics Approval

Ethics approval was received from the Dartmouth Hitchcock Medical Center institutional review board (study ID 29591). The pilot trial protocol is available by contacting the corresponding author. At the time of the study, a registry of pilot trials was not required. In addition, this was an open trial; thus, preregistration did not occur.

### Procedures

**Figure 1:**
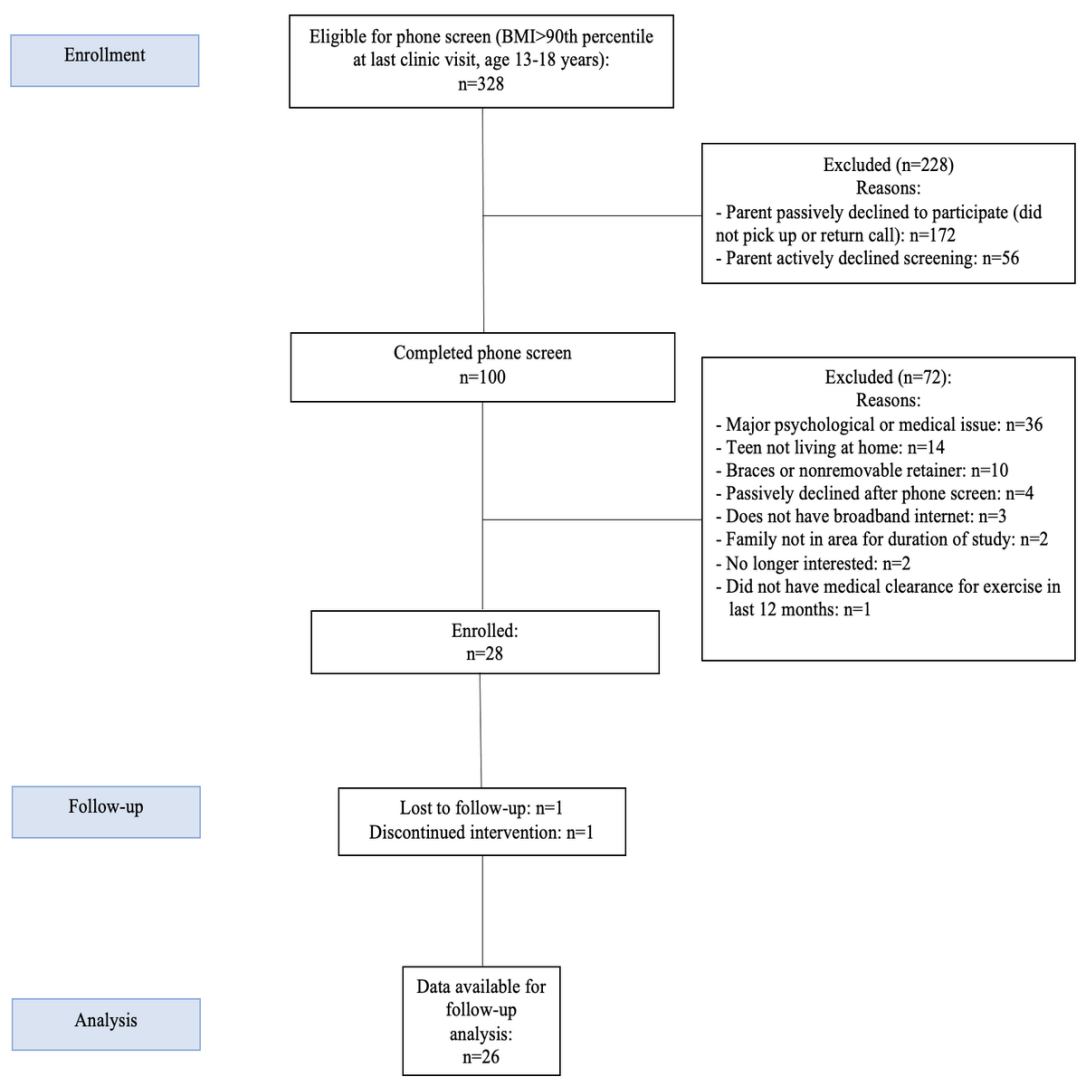
Participant Flow Chart.

A partial Health Insurance Portability and Accountability Act waiver was obtained, which allowed for the recruitment of participants from a general pediatric clinic at Dartmouth Hitchcock Medical Center using data from electronic medical records to identify potentially eligible adolescents based on age and BMI. Letters were sent to parents of potentially eligible adolescents, which provided instructions on how to opt out of the recruitment process (by calling a study coordinator within 2 weeks). After the 2-week opt-out period, research staff called families to tell them more about the study, assess their interest in participating, and determine eligibility (see Figure 1 for participant flow). Briefly, out of 100 adolescents who completed a phone screen, 28 (28%) adolescents enrolled in the study. Study recruitment took place between November 2016 and March 2017. Recruitment ended once 30% (30/100) of adolescents either enrolled or expressed strong interest in doing so. Intakes were conducted between December 2016 and March 2017. Participants completed the program on a rolling basis between winter 2016 and spring 2017. Follow-up assessments occurred throughout June 2017. Of the 28 participants, 26 (93%) completed their follow-up visits, 1 (3%) was lost to follow-up, and 1 (3%) withdrew from the study.

Informed consent and assent were obtained from parents and adolescents, respectively. All participants completed a baseline, in-person assessment that included a body composition assessment, web-based questionnaires, and computer tasks. Next, participants were provided a Fitbit Charge HR (Google LLC) to self-monitor their activity levels throughout the program. Participants then downloaded the Fitbit mobile application on their smartphone and created a Fitbit account, the credentials of which were shared with study staff for monitoring. The staff ensured that the Fitbit was set to track daily goals of 60 moderate to vigorous minutes, as recommended by the Centers for Disease Control and Prevention [26] and American Heart Association [27]. Although there is a lack of consistent activity guidelines, some prior studies have indicated that there is a correlation between 60 active minutes and a minimum of 10,000 steps in adolescents [3]. Therefore, step counts (≥10,000 steps) were included in the adolescents’ daily goals. Moreover, consistent with the self-determination theory, adolescent engagement in physical activity programs can be improved through supporting the basic psychological needs of adolescents, including autonomy [28]. Accordingly, the adolescents were given the opportunity to select whether to work toward their active minutes goal or their step goal each day. Adolescents were instructed on how to view daily progress toward exercise goals and incentives. Families were provided information on how to charge and wear the Fitbit (eg, wearing the device at all times when awake except when showering or otherwise exposed to water). Approximately 11% (3/28) of adolescents were loaned an iPod Touch as they did not have a smartphone on which to track their active minutes or receive daily text and email reminders.

An incentive program commenced on the first Monday following completion of the baseline visit to ensure consistency of feedback across participants. Table 1 shows program phases, weekly goals, and maximum incentives. Briefly, adolescents were provided a daily goal of >60 active minutes or 10,000 steps of exercise per day and weekly goals that depended upon the level of activity in the prior weeks. Adolescents were asked to sync their Fitbits at least once daily (either in the morning or at the end of the day) so that research staff could view their steps or active minutes from the previous day and to prevent loss of data (Fitbits store 30 days of data). Tuesday through Friday, texts informed adolescents about whether they met their active minute or step goal the previous day and praised them if they met their goal; if adolescents did not meet their goal or sync their Fitbit the previous day, texts reminded them to do so. In this pilot research phase, coaches only worked on weekdays; thus, feedback regarding goal success on Friday, Saturday, and Sunday were not delivered until the following Monday. Once per week texts on Monday informed adolescents about whether they met their *weekly* goal, praising them if they did and providing encouragement if they did not; these texts also informed adolescents of the amount of incentives earned that week, if applicable, and the goal for the upcoming week. SMS text message content was standardized and focused specifically on activity goals. However, if technical problems emerged with using or syncing the Fitbit, coaches provided support to adolescents and parents in navigating the technology. To provide immediate monetary reinforcement during the baseline and shaping phases, incentives for adherence to program weekly goals were loaded onto a reloadable debit card once per week for the first 9 weeks. To assess whether physical activity habits were formed rather than activity levels being contingent upon immediate monetary reinforcement, incentives for adherence to program weekly goals were only loaded at the end of the final week for the last 3 weeks in the program (ie, week 12, using a fading strategy).

Following the program, adolescents and parents attended a poststudy assessment and returned the Fitbit and Fitbit charger and iPod Touch if distributed. Adolescents earned US $100 for completing each assessment that included a lengthy functional magnetic resonance imaging session (US $200 total for baseline and poststudy assessment), although functional magnetic resonance imaging data were not analyzed in this study.

**Table 1:** Program phases, weekly goals, and maximum incentives (total US $510).

Week	Phase	Goal	Incentive if goal is met (US $)	Incentive if goal is exceeded (US $)	Incentives (US $ per week)	Total incentives paid (US $)
1	Baseline	Sync Fitbit ≥5 of 7 days to establish starting level for weekly goals	10	—^a^	10	10
2	Shaping	Individualized exercise goal: meet daily goal 1 day more than the prior week (maximum 5 days)	20	10	30	30
3	Shaping	If goal met, goal for next week increases by 1 day (maximum 5 days); if goal not met, new goal is to match prior week	25	10	35	35
4	Shaping	If goal met, goal for next week increases by 1 day (maximum 5 days); if goal not met, a new goal is to match prior week	30	10	40	40
5	Shaping	If goal met, goal for next week increases by 1 day (maximum 5 days); if goal not met, a new goal is to match prior week	35	10	45	45
6	Shaping	If goal met, goal for next week increases by 1 day (maximum 5 days); if goal not met, a new goal is to match prior week	40	10	50	50
7	Maintenance	≥60 minutes of exercise (or 10,000 steps), 5 days per week	40	10	50	50
8	Maintenance	≥60 minutes of exercise (or 10,000 steps), 5 days per week	40	10	50	50
9	Maintenance	≥60 minutes of exercise (or 10,000 steps), 5 days per week	40	10	50	50
10	Fading	≥60 minutes of exercise (or 10,000 steps), 5 days per week; incentives paid at the end of 3-week period	40	10	50	—
11	Fading	≥60 minutes of exercise (or 10,000 steps), 5 days per week; incentives paid at the end of 3-week period	40	10	50	—
12	Fading	≥60 minutes of exercise (or 10,000 steps), 5 days per week; incentives paid at the end of 3-week period	40	10	50	150

^a^Not available (weeks in which there was no weekly goal bonus or incentives were not delivered).

### Measures

#### Program Acceptability

Program acceptability was assessed via participant report of the helpfulness of the text-based coaching (ie, “How helpful did you find our texts?”), experience of using the Fitbit (ie, “How much did you enjoy wearing the Fitbit?”) and overall experience with the program (ie, “How did you like the program?”). Participants rated each question on a 7-item scale, with higher scores indicating more positive experiences. In addition, participants were given a Fitbit Charge HR to track their activity, including active minutes and steps. At the end of the intervention, participants indicated whether they would like to continue wearing the Fitbit after the end of the program.

#### Program Adherence

Program adherence was assessed via the percentage of days participants wore their Fitbit (ie, Fitbit adherence) and the number of weeks each participant met their goals and obtained bonuses. Accordingly, participants’ earning totals were considered as indicators of program adherence.

#### Physical Activity

As described above, all participants were provided with a Fitbit Charge HR as part of the program. Steps and active minutes were tracked while the Fitbit was worn. Fitbit’s algorithm considers active minutes to be those with ≥3 metabolic equivalents and only calculates active minutes after 10 minutes of continuous activity. Steps and active minutes were automatically summed daily. The Fitbit Charge has been used in prior studies to measure tracked activity, and a *valid* day of Fitbit use is often characterized as having at least 1500 steps tracked [29,30]. On the basis of this metric, in this study, with 2184 possible data points (26 participants × 84 days), 1989 (91.1%) days of activity tracking met this criterion.

#### Body Mass and Body Fat

A Tanita TBF-300A scale was used to measure body fat and weight at baseline and after the program. The Tanita scale uses bioelectrical impedance analysis, or gentle electric signals through the body, to measure body fat. The Tanita TBF-300A scale has been shown to be a reliable and valid measure of body fat [31,32] and weight [33]. Height was measured using a stadiometer. Data were collected by research staff once at the initial intake and once at the follow-up (post program) session. Data were manually documented in participants’ study records. According to the Freeman et al [34] equation, BMI and Centers for Disease Control and Prevention growth charts were used to compute the percentage of the 95th BMI percentile for age and sex.

### Analysis Plan

First, descriptive statistics were used to examine program acceptability and adherence. Second, to further characterize adherence to daily Fitbit use, correlations and two-tailed *t* tests were also used and compared the percentage of days participants wore their Fitbit based on sex, age, and percentage of the 95th BMI percentile for age and sex. Third, program adherence subgroups were identified, as determined by participants’ weekly activity goal success during the first and second half of the program. Fourth, to assess changes in tracked daily active minutes (dependent variable) and steps taken (dependent variable) during participation in the program, multilevel growth modeling was used. Time (ie, day 1 to day 84) was included as an independent variable. Due to the small sample size, demographic covariates were not included in the multilevel models. A separate model was computed for active minutes and steps. To improve model fit, for the model with steps as the dependent variable, the step count variable was transformed, with participants’ daily step count divided by 100. Full information maximum likelihood estimation was used to account for missing data (<10%). Finally, paired-sample *t* tests were used to examine changes in body mass (ie, percentage of the 95th BMI percentile for age and sex) and body fat from baseline to after the intervention. Change scores were used (ie, week 12 – week 1), with negative change scores indicating decreases in body mass and body fat.

## Results

### Program Acceptability

Full descriptive statistics of program acceptability are documented in Table 2. Briefly, participants rated the text-based coaching, Fitbit enjoyment, and overall program highly. In addition, out of 26 participants, 23 (85%) reported that they would like to continue to wear the Fitbit.

**Table 2:** Program acceptability and adherence.

Measures	Values, mean (SD)
How helpful were the SMS text messages? (scale of 1 to 7)	5.8 (1.3)
How much did you enjoy wearing the Fitbit? (scale of 1 to 7)	6.2 (1.3)
How did you like the program? (scale of 1 to 7)	6.6 (0.7)
Weeks (maximum of 11) that participants met the weekly goal	7.2 (3.5)
Weeks (maximum of 11) that participants met the weekly bonus	5.3 (3.8)
Total incentives (US $) earned during the program (maximum of US $500)	302.04 (163.85)

### Program Adherence

Full descriptive statistics of program adherence are documented in Table 2. Briefly, participants wore the Fitbit on 91.1% (SD 12.6%) of days on average, with a range of 85.2% (week 11) to 95.6% (week 4) and no notable decreases in Fitbit adherence from week 1 (92.9%) to week 12 (86.6%). There were no differences in the average percentage of days the Fitbit was worn based on sex (female: mean 87.99, SD 13.78; male: mean 93.33, SD 11.55; 2-tailed *t* test t_24_=−1.08; *P*=.29), age (*r*=−0.01; *P*=.97), or percentage of the 95th BMI percentile for age and sex (*r*=0.08; *P*=.72). Participants also met their weekly goals and bonus goals for an average of 7 (SD=3.5) and 5 (SD=3.8) of 11 possible weeks, respectively, each earning an average of approximately US $300 of the US $510 maximum.

Three program adherence subgroups were identified: sustained program adherence, nonsustained program adherence, and limited program adherence. Participants categorized as exhibiting sustained program adherence (11/26, 42%) included participants who met at least 80% of their weekly goals across the full program period (ie, weeks 2 to 12). Participants categorized as demonstrating nonsustained program adherence (7/26, 27%) included participants who met 80% of their weekly goals in the first half of the program during shaping (ie, weeks 2 to 6) but not during maintenance and fading (ie, weeks 7 to 12). Participants categorized as showing limited program adherence (7/26, 27%) included participants who did not meet 80% of their weekly goals during either half of the program. Of the 28 participants, 1 (4%) exhibited delayed program adherence (ie, met 80% of weekly goals only during maintenance and fading).

### Physical Activity

Findings demonstrated a significant increase in tracked daily active minutes (*P*=.006) and steps (*P*<.001), with an average increase of 20.41 (SD 34.27) tracked active minutes per day and 924.00 (SD 2141.59) tracked steps per day found across the entire intervention. There was also a significant intercept by slope effect in the daily active minutes multilevel model (*P*=.005), indicating that adolescents who demonstrated greater active minutes per day at the start of the intervention also demonstrated the greatest increases in active minutes per day over the full intervention period. Full test statistics are presented in Tables 3-6.

**Table 3:** Changes in daily active minutes (fixed effects).

Fixed effects			*b* (SE; 95% CI)			*t* test		*P* value	
Intercept			54.80 (6.28; 67.10-42.50)			8.73		<.001	
**Within-subjects (level 1)**									
	Time	0.24 (0.08; 0.40-0.08)				2.96		.003	

**Table 4:** Changes in daily active minutes (random effects).

Random effects (covariances)			*b* (SE; 95% CI)			Wald *Z*		*P* value	
**Within-subjects**									
	Residual	3113.47 (100.04; 3309.56-2917.39)				31.12		<.001	
**Between-subjects**									
	Intercept	859.48 (283.12; 1414.39-304.57)				3.04		.002	
	Time	0.10 (0.05; 0.20-0.01)				2.15		.03	
	Intercept and time	7.98 (2.82; 13.51-2.45)				2.83		.005	

**Table 5:** Changes in daily steps (fixed effects).

Fixed effects		*b* (SE; 95% CI)	*t* test	*P* value
Intercept		93.13 (3.84; 100.64-85.61)	24.28	<.001
**Within-subjects (level 1)**				
	Time	0.11 (0.05; 0.22- 0.01)	2.05	.04

**Table 6:** Changes in daily steps (random effects).

Random effects (covariances)		*b* (SE; 95% CI)	Wald *Z*	*P* value
**Within-subjects**				
	Residual	1129.73 (36.32; 1200.91 to 1058.55)	31.11	<.001
**Between-subjects**				
	Intercept	322.18 (106.89; 531.69 to 112.68 )	3.01	.003
	Time	0.05 (0.02; 0.09 to 0.01)	2.30	.02
	Intercept and time	0.42 (0.10; 2.57 to –1.74)	0.38	.71

### Body Mass and Body Fat

Findings demonstrated a significant decrease in body fat percentage from week 1 to week 12 (*P=*.04). Change in percentage of the 95th BMI percentile for age and sex was nonsignificant (*P*>.05). Full test statistics are documented in Table 7.

**Table 7:** Descriptive statistics for physical health outcomes at baseline to 12-week follow-up for the entire sample.

Outcomes	Baseline, mean (SD)	Follow-up, mean (SD)	*t* test	*P* value
Percentage of the 95th percentile for age and sex	110.20 (11.42)	110.46 (10.33)	–0.26	.80
Body fat (%)	35.54 (7.79)	34.61 (8.46)	2.17	.04

## Discussion

### Principal Findings

This was a single group pre–post pilot study testing a novel mHealth physical activity program for adolescents with overweight and obesity. Despite the lack of a control group, this study provides preliminary support that an mHealth program with incentives and text-delivered support and feedback may be an effective nonintensive alternative for increasing tracked physical activity and reducing body fat in adolescents with overweight and obesity. Adolescents reported a positive experience with each program component (ie, text-based coaching and use of Fitbit) and with the program as a whole. There was a low attrition rate (2/28, 7%). These indicators of program acceptability were consistent with program adherence data that showed that participants wore their Fitbit over 90% of program days, and Fitbit adherence was consistent across sex, age, and percentage of the 95th BMI percentile for age and sex groups. In addition, most participants regularly met their weekly goals, especially in the shaping period of the program from weeks 1 to 6, during which 64.3% (18/28) of participants met at least 80% of their weekly goals. In addition, participants acquired bonuses for exceeding their predetermined goals in about half of the total weeks (5.3 of 11 possible weeks), suggesting positive program outcomes. Across all enrolled participants, the program led to significant improvements in tracked time spent engaging in daily active minutes with metabolic equivalents ≥3 and steps, as well as significant reductions in body fat percentage, although no significant changes in body mass were noted. Notably, these changes in health and health behavior were achieved with minimal human resources (eg, human contact involved 1 brief in-person *start-up* meeting with a bachelor’s level coach and then 5 brief SMS text messages each week, with incentives delivered electronically).

Intensive, multi-component behavior modification studies for health behavior change have faced challenges in achieving high adolescent adherence and have struggled to maintain adolescent motivation throughout the program [9,35]. However, within this study, we had limited attrition, and participants reported favorable opinions of the program, including Fitbit use. Participants were also highly engaged, often meeting their activity goals—especially in the first half of the intervention—and wearing their Fitbit regularly. A reason for the promising level of adherence found within this program may be the low burden of participation combined with incentives. In addition, the use of sensor data collection, in lieu of self-report measures of physical activity, is likely less burdensome and provides a more objective and continuous measure of physical activity [36]. This low level of burden can be contrasted with multi-component intervention programs for treating overweight or obesity, which often involve parental engagement, family therapy, and nutritional changes [11,37]. The findings of the study are promising in that they suggest that an mHealth program with remote incentives, goal setting, and text-delivered support is acceptable within an adolescent sample.

Significant improvements in tracked daily active minutes and steps were also found, highlighting the promise of the program in facilitating relatively immediate health behavior change to support adolescents to manage overweight and obesity. Notably, participants demonstrated an average increase of >20 tracked active minutes per day and 924 tracked steps per day over the course of the 12-week program. Recent research suggests a 20-minute increase in activity per day in adults would lead to a 13% decrease in the number of deaths per year [38]. Therefore, the demonstrated physical activity improvements in response to this novel program are noteworthy and further underscore the feasibility of the program in evoking health behavior change in this high-risk population.

Although some research has shown that increased physical activity predicts decreased body mass [39], this study observed no change in percentage of the 95th BMI percentile for age and sex. This finding is more consistent with well-established weight loss research indicating that dietary changes rather than physical activity alone drive weight loss among individuals with overweight and obesity [40,41]. Nevertheless, participants demonstrated an overall decrease in body fat, underscoring the ways in which improved tracked physical activity improves physical health [42]. Dissemination of this type of physical activity program may reduce the risk of overweight and obesity, as well as of endocrine and cardiovascular diseases [43], in adulthood.

### Limitations

The findings of the study should be interpreted in the context of its limitations. There was no control group in the study; thus, the effects found in the program cannot be attributed to the program alone. In addition, the sample size was small, and there were subgroups with varying mean levels of adherence, highlighting the need to further modify the program to address nonresponse in some participants. In addition, the reduction in body fat, although statistically significant, may not be clinically significant. In addition, participants were primarily White, and only a paucity of participants reported having public insurance, of which the latter is a strong indicator of low socioeconomic status (SES); thus, the findings might not be generalizable to racially or ethnically diverse samples and adolescents from low SES backgrounds. In addition, the sample includes adolescents at or above the 90th BMI percentile, such that the generalizability of findings to those adolescents with overweight but between the 85th and 89.9th BMI percentile is unknown. Although there was an even distribution of girls and boys, an assessment of differences in changes in physical activity and physical health based on sex could not be conducted. Notably, pubertal status was not assessed in this study, and pubertal changes over the course of the program may have affected physical health results.

In addition, it is possible that participants who enrolled in the study were more motivated to increase physical activity than those who declined. Nonetheless, we used universal outreach (ie, completed a chart review to identify potentially eligible participants) to identify those individuals who may be eligible but otherwise were not seeking behavioral care for the management of overweight or obesity. In addition, owing to the novelty of the Fitbit and monitoring of activity by the text-based health coach, there may have been an increase in activity during the baseline period relative to *usual* activity levels, and the true baseline level of activity before receiving the Fitbit was unknown. However, such an impact would lessen the increase in activity over the course of the intervention.

There also may have been measurement errors, as a recent meta-analysis indicated that Fitbit estimates of step count and moderate to vigorous active minutes might be less accurate than research-grade accelerometers [44], and there was no blinded research-grade measurement of activity before, during, and after the intervention. Regarding the physical health measures, there may have been measurement error in the body mass and body fat calculations, as participants were not given specific instructions to fast or avoid fluid intake before the physical health assessments, and all measurements were taken only once at each study time point.

There are also limitations in the program structure. First, owing to the lack of a universal guideline, participants were given a 10,000 daily step goal, which is lower than the physical activity recommendations used in other studies (eg, 12,000 daily step count, which is more strongly correlated with 60 active minutes) [45]. Second, long-term follow-up assessments were not conducted. In addition, as there were multiple treatment components, it is unknown which specific components guided program effects and whether the program would be as effective with even fewer components. Finally, participants were eligible to receive up to US $510 in incentives, which may limit implementation by some health systems. It is important to note that participants had an average weekly earning of US $25.34, which equated to only approximately 59% of the maximum possible incentive earnings (ie, average incentives earned per participant was only US $302.04 across the entire 12-week program). The scalable program yielded a more optimal cost-effectiveness ratio than traditional, multi-component in-person interventions [46].

### Conclusions and Future Directions

This pilot study provides preliminary support of the acceptability and feasibility of a remote, mHealth program comprising tracked physical activity goals, incentives, and text-based support from health coaches to improve tracked physical activity and reduce body fat in adolescents with overweight and obesity. Replication of findings in a randomized trial by involving a larger, more diverse sample is warranted, including youth with BMI percentiles >85th, and may further clarify the utility of the program in addressing the pediatric overweight and obesity epidemic. Importantly, future studies should explore individual-level factors (eg, sex, cultural background, SES, motivation to lose weight, and pubertal status) that may predict program adherence and changes in tracked activity levels, as well as examine whether program effects are *clinically* significant. To allow for comparison of findings across studies, it should also be a priority to establish a universal cutoff for Fitbit wear time (or adherence), including the minimum number of steps and minutes required to be considered a full day of wear time, as well as universal guidelines for daily step and active minutes for adolescents, specifically. Future studies could investigate the incremental efficacy of each program component using a factorial design to compare the efficacy of an SMS text message only program, incentives only program, and SMS text message plus incentives program in increasing physical activity among adolescents with overweight or obesity. Researchers might also consider investigating the differential effects of varying incentive magnitudes on changes in tracked physical activity. Given that health insurers are poised to deliver incentives for health behavior change, a randomized trial of this program could advance the evidence-based use of incentives by health insurers. Researchers may also examine whether other incentive types, including parent-led behavior contracting and provision of rewards other than incentives (eg, time with peers and low-cost books or games), may evoke similar results. An adaptive program might also be tested, in which participants are initially offered smaller incentives for a brief period, with participants who demonstrate limited program adherence switching to a more intensive program (eg, parental involvement and larger incentives). In addition, the text-based mHealth program used in this study could be automated by using technology such as the Fitbit application programming interface to automate goal setting and deploy reinforcing SMS text messages every day. The program could also be lengthened to support greater reductions in body fat, as well as be modified to target only increased active minutes or only step counts. Importantly, all future iterations of the program should implement an intervention mapping framework [47].

Future research may also consider assessing mediators and moderators of change. Other adaptions that might be tested include personalized daily adaptive goals (in addition to weekly goals) and incentives for meeting daily goals to increase the number of adolescents meeting weekly goals. In addition, a longer fading period (eg, 12 weeks of weekly incentives, followed by 4-6 weeks of more gradual fading) may support sustained physical activity improvements and long-term program response. Finally, future research should investigate whether participation in an incentive program may increase motivation for additional lifestyle behavior changes (eg, diet and sleep patterns) associated with overweight and obesity, as well as guide the long-term prevention of having overweight and obesity and their common comorbidities during adulthood. Combined, such avenues for future program development may allow for a highly scalable, accessible, and sustainable program for adolescents with overweight and obesity.

## Abbreviations

mHealth: mobile health
PI: principal investigator
SES: socioeconomic status
